# Risk factors for gestational diabetes: An umbrella review of meta-analyses of observational studies

**DOI:** 10.1371/journal.pone.0215372

**Published:** 2019-04-19

**Authors:** Konstantinos Giannakou, Evangelos Evangelou, Panayiotis Yiallouros, Costas A. Christophi, Nicos Middleton, Evgenia Papatheodorou, Stefania I. Papatheodorou

**Affiliations:** 1 Cyprus International Institute for Environmental & Public Health, Cyprus University of Technology, Limassol, Cyprus; 2 Department of Hygiene and Epidemiology, University of Ioannina, School of Medicine, University Campus, Ioannina, Greece; 3 Department of Epidemiology and Biostatistics, School of Public Health, Imperial College London, Norfolk Place, London, United Kingdom; 4 Medical School, University of Cyprus, Nicosia, Cyprus; 5 Department of Nursing, School of Health Sciences, Cyprus University of Technology, Limassol, Cyprus; 6 “Kentro Iatrikis Ioanninon”, Ioannina, Greece; 7 Department of Epidemiology, Harvard TH Chan School of Public Health, Boston, Massachusetts, United States of America; University of California Los Angeles, UNITED STATES

## Abstract

**Background/Objective:**

Gestational diabetes mellitus (GDM) is a common pregnancy complication, with complex disease mechanisms, and several risk factors may contribute to its onset. We performed an umbrella review to summarize the evidence from meta-analyses of observational studies on risk factors associated with GDM, evaluate whether there are indications of biases in this literature and identify which of the previously reported associations are supported by convincing evidence.

**Methods:**

We searched PubMed and ISI Web of Science from inception to December 2018 to identify meta-analyses examining associations between putative risk factors for GDM. For each meta-analysis we estimated the summary effect size, the 95% confidence interval, the 95% prediction interval, the between-study heterogeneity, evidence of small-study effects, and evidence of excess-significance bias.

**Results:**

Thirty eligible meta-analyses were identified, providing data on 61 associations. Fifty (82%) associations had nominally statistically significant findings (P<0.05), while only 15 (25%) were significant at P<10^−6^ under the random-effects model. Only four risk factors presented convincing evidence:, low vs. normal BMI (cohort studies), BMI ~30–35 kg/m2 vs. normal BMI, BMI >35 kg/m2 vs. normal BMI, and hypothyroidism.

**Conclusions:**

The compilation of results from synthesis of observational studies suggests that increased BMI and hypothyroidism show the strongest consistent evidence for an association with GDM. Diet and lifestyle modifications in pregnancy should be tested in large randomized trials. Our findings suggest that women with known thyroid disease may be offered screening for GDM earlier in pregnancy.

## Introduction

Gestational diabetes mellitus (GDM) is a common pregnancy complication, defined as glucose intolerance with onset or first recognition during pregnancy, in women without prior diabetes history prior to pregnancy.[1, 2] During the last 20 years the prevalence of GDM has increased worldwide and it is expected to continue to rise along with the increase in pre-conception obesity and pregnant women affected by obesity.[3] GDM affects approximately 15% of all pregnancies, depending on population characteristics, and this prevalence may in fact be higher under the new diagnostic criteria.[4, 5] GDM is associated with an increased risk of maternal and infant morbidity, including macrosomia, large for gestational age (LGA), cesarean section delivery and preterm birth, but it is also considered to be a risk factor for long-term complications, such as type 2 diabetes mellitus and cardiovascular disease in the mother and the offspring.[6–9] The etiology of GDM is multifactorial and has not completely been established yet, while several risk factors may contribute to its onset. Age, overweight or obesity, ethnicity, family history of diabetes, and history of GDM are some of the proposed risk factors for GDM.[10–13]

Meta-analyses of randomized clinical trials for GDM prevention that evaluated a range of dietary and lifestyle interventions during pregnancy, including diet and exercise, lifestyle advice, nutritional manipulation, and behavior modification, showed inconsistent findings, with some meta-analyses reporting significant deceased incidence of GDM [14–18], while others were null. [19–24]

Under the prism of the abundance of observational significant associations, we conducted an umbrella review of meta-analyses on risk factors for GDM. Using a standardized approach, we aimed to assess the credibility of those findings to identify which associations are with robust epidemiological evidence.

## Methods

### Search strategy

This study was performed according to the guidelines for systematic reviews under the Preferred Reporting Items for Systematic Reviews and Meta-Analyses (PRISMA).[25]

We conducted an umbrella review, which is a systematic collection and evaluation of multiple systematic reviews and meta-analyses performed on a specific research topic.[26] An umbrella review examines comparisons of a large number of existing systematic reviews and meta-analyses on risk factors into one accessible and usable document.[26, 27] The methods of performing an umbrella review are standardized and, in this work, we followed the same principles used in previously published umbrella reviews across various fields of research.[28–31] We used a ranking system to grade the evidence from meta-analyses of observational studies in terms of the significance of the summary effect, 95% prediction interval, presence of large heterogeneity, small study effects, and excess significance bias.

Two researchers (KG and SP) independently searched PubMed and ISI Web of Science from inception to 23 of December 2018 to identify meta-analyses of observational studies examining associations regarding risk factors for GDM. The search strategy used the keywords (“gestational diabetes” OR “pregnancy diabetes” OR “pregnancy hyperglycemia” OR “3 h abnormal gtt test” OR “insulin during pregnancy” OR “antidiabetics during pregnancy” OR “metformin in pregnancy”) AND (“systematic review” OR “meta-analysis”). All identified publications went through a three-step parallel review of title, abstract, and full text, performed by KG and SP, based on predefined inclusion and exclusion criteria. We also screened the references of the retrieved articles for possible eligible papers. Any disagreement was resolved with discussion.

We included meta-analyses of observational studies (i.e., cross-sectional, case-control and cohort studies), which investigated risk factors for GDM. Meta-analyses were retained if they included at least three studies in which information was provided per included study on a measure of association, its standard error, the number of cases and the total population. We did not apply any language restrictions in the selection of eligible studies. We included only meta-analyses of epidemiological studies in humans. We excluded studies in which risk factors were used for screening, diagnostic, or prognostic purposes, or meta-analyses that examined GDM as a risk factor for other medical conditions. We also excluded studies on women with pre-existing type II diabetes. We excluded systematic reviews and meta-analyses of genetic risk factors, narrative reviews, letters to the editor, meta-analyses of Randomised Control Trials (RCTs), and systematic reviews without a quantitative synthesis of data. If an article presented meta-analyses on other pregnancy outcomes including GDM, we only extracted information on the latter. When more than one meta-analysis on the same research question was eligible, the meta-analysis with the largest number of component studies with data on individual studies’ effect sizes was retained for the main analysis to avoid duplication of the study populations.

### Data extraction

Data extraction was performed independently by two investigators (KG, SP), and in case of discrepancies, the final decision was reached by consensus, involving a third investigator, when necessary (EE). From each eligible meta-analysis, we extracted information on the first author, year of publication, the examined risk factors, the number of studies included, the study-specific relative risk estimates (risk ratio, odds ratio, or standardized mean differences) along with the corresponding confidence intervals (CI). Also, we recorded the reported summary meta-analytic estimates using both fixed and random effect methods along with the corresponding confidence intervals, the total population, and number of cases for each study. We also recorded whether the selected meta-analyses applied any criteria to evaluate the quality of the included studies.

### Statistical analysis

For each meta-analysis, we re-calculated the summary effect and its 95% CI by using both fixed and random effect models.[32, 33] We also calculated the 95% prediction intervals (PI) for the summary random effects estimates, which further accounts for between-study heterogeneity and indicates the uncertainty for the effect that would be expected in a new study addressing the same association.[34, 35] We considered the largest study as the most precise with a difference between the point estimate and the upper or lower 95% confidence interval less than 0.20 (characterized as small effect size for a continuous outcome according to Cohen’s d definition.[36] We also recorded whether the largest study presented a statistically significant effect as part of the grading criteria.

We assessed heterogeneity among studies, and we reported the P value of the χ^2^-based Cochran Q test and the I^2^ metric for inconsistency, which could reflect either diversity or bias. I^2^ metric ranges between 0% and 100% and quantifies the variability in effect estimates that is due to heterogeneity rather than sampling error.[37] Values exceeding 50% or 75% are usually considered to represent large or very large heterogeneity, respectively. Confidence intervals were calculated as per Ioannidis et al.[38]

Moreover, we assessed whether there is evidence for small study effect meaning whether smaller studies tend to give substantially larger estimates of effect size compared with larger studies. Small study effects can indicate publication and other selective reporting biases, but they can also reflect genuine heterogeneity, chance, or other reasons for differences between small and large studies.[39] We used the regression asymmetry test proposed by Egger et al for this assessment.[40] A P value <0.10 with more conservative effect in larger studies was considered evidence of small-study effects.

We further applied the excess significant test to evaluate whether there is a relative excess of significant findings in published literature due to any reason (e.g. publication bias, selective reporting of outcomes or analyses). This is a chi-squared-based test, in which the number of expected positive studies is estimated and compared against the number of observed number of studies with statistically significant results (P<0.05).[41] A binomial test was then used to evaluate whether the number of positive studies in a meta-analysis is too large according to the power that these studies have to detect plausible effects at α = 0.05. Briefly, a comparison between observed vs. expected is performed separately for each meta-analysis and it is also extended to research areas of many meta-analyses after summing the observed and expected from each meta-analysis. The expected number of significant studies for each meta-analysis is calculated by the sum of the statistical power estimates for each component study.[41] The power of each component study was estimated using the fixed or random effects summary, or the effect size of the largest study (smallest SE) as the plausible effect size.[42] The power of each study was calculated with an algorithm using a non-central t distribution.[43] Excess statistical significance for single meta-analyses was claimed at P<0.10 (one-sided P<0.05, with observed > expected as previously proposed).[41] We classified risk factors into categories based on biological pathways or types of exposures involved: biomarkers, nutrition and lifestyle factors, diseases and disorders, infections, and other factors. We examined excess of statistical significance separately in each of these categories as selective reporting bias may arise in different categories of research.

### Assessment of epidemiologic credibility

We characterized as convincing the associations fulfilling the following criteria: a significant effect under the random-effects model at P<10^−6^ [44, 45], more than 1000 cases, between-study heterogeneity was not large (I^2^<50%), the 95% PI excluding the null value, and no evidence of small-study effects or excess of significance bias. Additionally, associations with more than 1000 cases, a significant effect at P<10^−6^, and a nominally statistically significant effect present at the largest study were characterized as highly suggestive. We considered as suggestive the associations with significant effect at P<10^−3^ and more than 1000 cases. The remaining statistically significant associations at P<0.05 under random-effects model were graded as weak associations.

Two independent investigators (KG, SP) assessed the methodological quality of all included systematic reviews and meta-analyses of observational studies using the Assessment of Multiple Systematic Reviews (AMSTAR) tool.[46] The AMSTAR is an 11-item instrument with scores ranging from 0 to 11 related to vital features of the methodological rigor across systematic reviews and meta-analyses with higher scores indicating greater quality. AMSTAR scores are graded as high (8–11), medium (4–7), and low quality (0–3).[46, 47]

All authors had full access to all the data in the study. Statistical analyses were performed in STATA version 14 (STATA Corp, College Station, TX).

## Results

### Description of eligible meta-analyses

**Fig 1:**
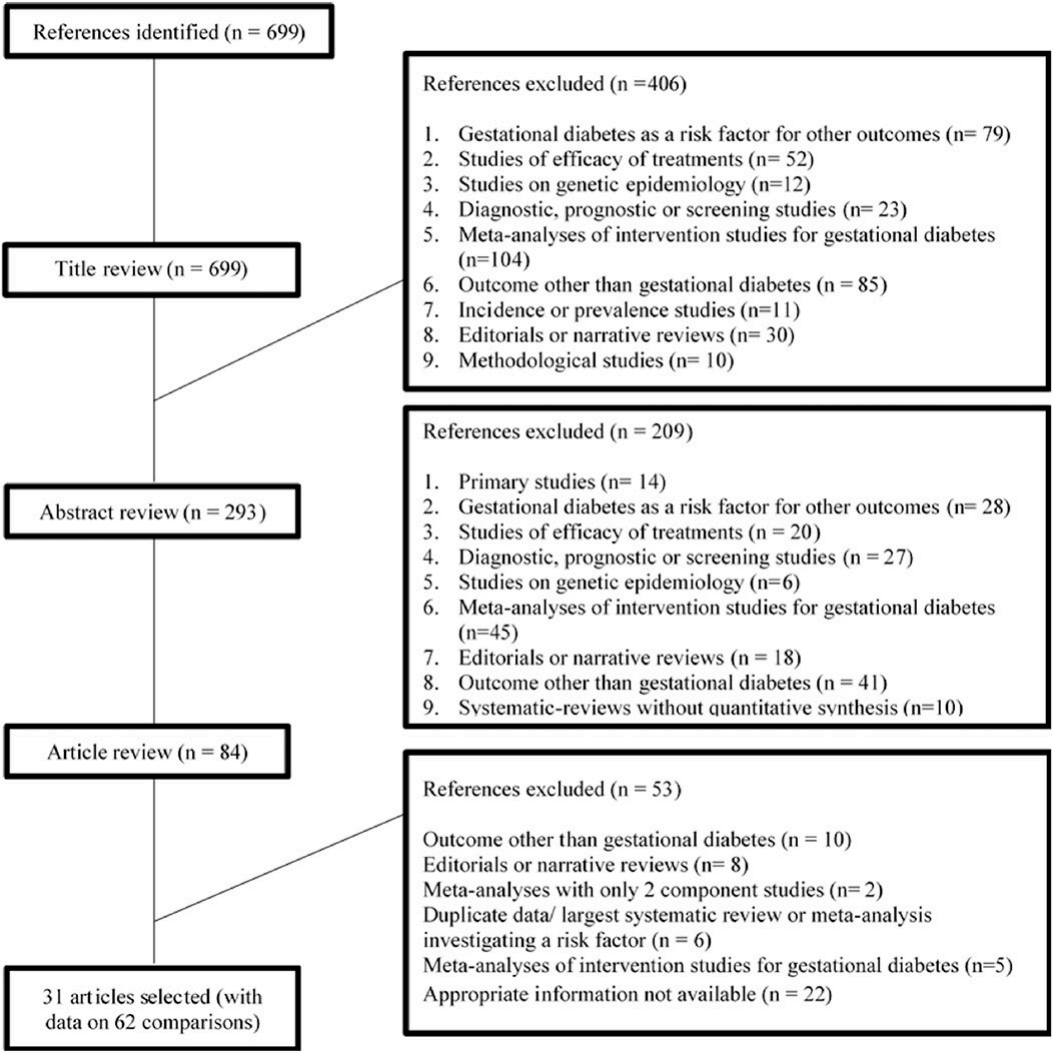
Flow diagram for the selection of included articles.

Overall, the literature search identified 699 publications of which 616 were excluded after the title and abstract review. Of the 83 articles screened in full text, 22 articles did not report the appropriate information for the calculation of excess of statistical significance (either because the total sample size was missing or the study-specific relative risk estimates were missing), 10 articles were excluded because the outcome of interest was not gestational diabetes, 8 because were editorials or narrative reviews, 5 because were meta-analyses of RCTs, 6 articles excluded because a larger systematic review or meta-analysis including all previous studies investigating the same risk factor was available, and 2 articles were excluded because included only 2 component studies (Fig 1). The 30 eligible papers [17, 48–76] included data on 61 different meta-analyses (comparisons) in five broad areas (biomarkers [n = 23 comparisons], nutrition and lifestyle [n = 20 comparisons], diseases and disorders [n = 8 comparisons], infections [n = 2 comparisons], and other factors [n = 8 comparisons]). There were 3 to 40 studies per meta-analysis, with a median of 9 studies. The publication date of the eligible articles ranged between 2009 and 2018. The median number of case and control participants in each study was 84 and 325, respectively. The median number of case and control subjects in each meta-analysis was 1747 and 13850, respectively. The number of cases was greater than 1000 in 38 (62%) meta-analyses (Table 1).

**Table 1:** Quantitative synthesis and assessment of bias across the 61 associations of risk factors for gestational diabetes.

Area	Author, year	Comparison	Studies	Cases/controls	Random effects*	Largest effect‡	P Random	Egger§	I^2^ (P)||	95% PI ≠
Biomarkers	Zhou Z 2018	Chemerin levels	13	742/840	5.40 (1.28–22.8)	127.8 (82–199.1)	0.0220	0.591	97 (<0.001)	0.02–1863
Biomarkers	Zhang W 2018	Visfatin levels	26	1033/1272	1.57 (0.86–2.83)	2.34 (1.54–3.55)	0.1387	0.764	92 (<0.001)	0.07–33.76
Biomarkers	Kataria Y 2018	Ferritin concentration (ng/mL)	12	2152/46443	16.38 (2.77–96.9)	1.36 (1.05–1.77)	2.1 x 10^−3^	0.108	99 (<0.001)	0.01–23068
Biomarkers	Kataria Y 2018	Hemoglobin concentration (g/dL)	9	1022/3531	4.34 (2.07–9.08)	1.39 (1.05–1.83)	9.9 x 10^−5^	0.013	96 (<0.001)	0.30–62.4
Biomarkers	Zhou Z 2018	Mean platelet volume	20	1466/1951	4.09 (2.24–7.47)	0.67 (0.51–0.89)	4.35 x 10^−6^	0.017	95 (<0.001)	0.24–69.6
Biomarkers	Amraei M 2018	Insufficient vitamin D	26	5464/15039	1.39 (1.18–1.63)	1.58 (0.85–3.78)	5.5 x 10^−5^	0.068	43 (0.011)	0.80–2.41
Biomarkers	Amraei M 2018	Serum 25(OH)D level	16	1337/4158	0.62 (0.49–0.78)	0.56 (0.34–0.93)	5.9 x 10^−5^	0.681	69 (<0.001)	0.26–1.46
Biomarkers	Tiongco RE 2018	Maternal iron deficiency	6	358/14799	0.61 (0.47–0.80)	0.80 (0.32–1.99)	2.9 x 10^−4^	0.864	0 (0.687)	0.42–0.89
Biomarkers	Kong FJ 2017	Betatrophin levels	8	401/421	6.65 (2.12–20.9)	16.5 (9.18–29.8)	1.17 x 10^−3^	0.191	94 (<0.001)	0.11–411.7
Biomarkers	Fu S 2016	Ferritin (highest vs lowest ferritin levels) (cohorts)	4	214/1662	3.22 (1.73–6.00)	4.98 (1.46–17.03)	2.37 x 10^−4^	0.953	0 (0.815)	0.82–12.65
Biomarkers	Fu S 2016	Serum ferritin (GMD-women vs non-GMD)	6	403/498	4.89 (2.06–11.58)	6.45 (4.07–10.24)	3.10 x 10^−4^	0.756	91 (<0.001)	0.22–106.6
Biomarkers	Fernández-Cao JC 2016	Hemoglobin levels (highest vs lowest levels)	9	792/4393	1.54 (1.18–2.03)	0.81 (0.36–1.82)	1.80 x 10^−3^	0.752	33 (0.157)	0.81–2.93
Biomarkers	Fernández-Cao JC 2016	Ferritin (highest vs lowest levels)	7	330/5574	2.09 (1.48–2.96)	2.27 (1.20–4.30)	3.27 x 10^−5^	0.600	1 (0.42)	1.31–3.34
Biomarkers	Hu S 2016	Serum retinol-binding protein-4	17	647/620	4.38 (2.10–9.14)	1.27 (0.70–2.30)	8.47 x 10^−5^	0.025	91 (<0.001)	0.18–106.7
Biomarkers	Guo CC 2016	DQ2	12	2333/2687	1.36 (1.10–1.66)	0.96 (0.79–1.16)	3.65 x 10^−3^	0.008	43 (0.06)	0.80–2.30
Biomarkers	Guo CC 2016	DQ6	11	2270/2576	0.81 (0.69–0.94)	0.75 (0.55–1.02)	7.56 x 10^−3^	0.551	0 (0.743)	0.67–0.97
Biomarkers	Guo CC 2016	DR 13	4	209/225	2.46 (1.02–5.90)	0.73 (0.29–1.87)	.04437	0.982	67 (0.03)	0.07–88.5
Biomarkers	Guo CC 2016	DR17	5	329/335	3.16 (1.31–7.64)	3.13 (1.11–8.81)	.01054	0.116	69 (0.01)	0.16–62.9
Biomarkers	Yang Y 2015	Thyroid antibodies (cohort)	11	1596/30012	1.07 (0.97–1.19)	1.18 (0.77–1.81)	.19124	0.546	0 (0.44)	0.95–1.21
Biomarkers	Yang Y 2015	Thyroid antibodies (case-control)	10	856/2062	1.21 (1.05–1.41)	1.33 (1.09–1.63)	.01042	0.402	0 (0.73)	1.02–1.44
Biomarkers	Yang Y 2015	Thyroid antibodies (All studies)	21	2452/32074	1.12 (1.03–1.22)	1.18 (0.77–1.81)	.01065	0.485	0 (0.60)	1.02–1.23
Biomarkers	Wei SQ 2013	25(OH)D5<50 nmol/l	10	623/3503	1.37 (1.11–1.70)	1.20 (0.72–2.00)	3.18 x 10^−3^	0.147	0 (0.51)	1.07–1.76
Biomarkers	Wei SQ 2013	25(OH)D<75 nmol/l	8	542/3298	1.52 (1.17–1.98)	1.63 (0.79–3.33)	1.64 x 10^−3^	0.954	7 (0.37)	1.01–2.30
Nutrition and lifestyle	Najafi F 2018	Pre-pregnancy BMI (as a continuous variable)	5	1605/3112	1.19 (1.13–1.26)	1.16 (1.14–1.18)	3.53 x 10^−10^	0.499	82 (<0.001)	0.98–1.44
Nutrition and lifestyle	Davenport 2018	Prenatal exercise + co-interventions	4	81/265	0.47 (0.25–0.89)	0.41 (0.20–0.86)	0.020	0.719	16 (0.31)	0.08–2.92
Nutrition and lifestyle	Davenport 2018	Prenatal exercise (cohort studies)	14	343/9252	0.68 (0.53–0.87)	0.59 (0.43–0.82)	0.002	0.259	0 (0.62)	0.52–0.89
Nutrition and lifestyle	Davenport 2018	Prenatal exercise (cross-sectional studies)	8	136/5504	0.66 (0.44–0.97)	0.63 (0.32–1.26)	0.03	0.232	0 (0.69)	0.40–1.07
Nutrition and lifestyle	Davenport 2018	Prenatal exercise (case-control studies)	4	196/451	0.54 (0.23–1.27)	0.99 (0.60–1.62)	0.544	0.367	62 (0.05)	0.02–13.6
Nutrition and lifestyle	Fu S 2016	Dietary total iron intake	3	1007/13850	1.01 (1.00–1.01)	1.12 (0.87–1.45)	2.78 x 10^−8^	NA	0 (0.73)	0.99–1.03
Nutrition and lifestyle	Kong FJ 2016	Selenium level	7	178/391	0.12 (0.03–0.53)	0.12 (0.06–0.26)	5.00 x 10^−3^	0.499	93 (<0.001)	0.00–19.81
Nutrition and lifestyle	Aune D 2016	Leisure-time physical activity before pregnancy	8	2401/30191	0.78 (0.61–1.00)	0.81 (0.68–1.01)	.05027	0.869	47 (0.07)	0.41–1.47
Nutrition and lifestyle	Aune D 2016	Leisure-time physical activity during pregnancy	5	580/5140	0.97 (0.73–1.28)	0.91 (0.37–2.21)	.81601	0.430	0 (0.80)	0.61–1.52
Nutrition and lifestyle	Torloni MR 2009	Low vs. Normal BMI (cohort)	16	75669/280734	0.75 (0.69–0.83)	0.80 (0.69–0.92)	1.55 x 10^−9^	0.022	16 (0.27)	0.63–0.90
Nutrition and lifestyle	Torloni MR 2009	Low vs. Normal BMI (case-control)	3	5957/11651	0.65 (0.51–0.83)	0.61 (0.47–0.81)	4.47 x 10^−4^	0.572	0 (0.83)	0.13–3.16
Nutrition and lifestyle	Torloni MR 2009	Overweight vs. Normal BMI (cohort)	17	112880/282458	1.97 (1.76–2.19)	2.29 (2.12–2.47)	8.01 x 10^−35^	0.521	56 (0.003)	1.44–2.68
Nutrition and lifestyle	Torloni MR 2009	Overweight vs. Normal BMI (case-control)	3	287/501	2.68 (1.78–4.04)	3.85 (2.30–6.47)	2.33 x 10^−6^	0.889	40 (0.19)	0.05–138
Nutrition and lifestyle	Torloni MR 2009	Obese (BMI >30) vs. normal weight	31	56333/308335	3.76 (3.31–4.28)	4.80 (4.43–5.21)	0	0.661	73 (<0.001)	2.23–6.34
Nutrition and lifestyle	Torloni MR 2009	Obese 1 (BMI ~30–35) vs. Normal weight	6	3087/20901	3.01 (2.34–3.86)	3.21 (2.68–3.85)	8.88 x 10^−18^	0.612	27 (0.23)	1.71–5.28
Nutrition and lifestyle	Torloni MR 2009	Obese 2 (BMI >35) vs. Normal weight	7	1747/21001	5.52 (4.28–7.11)	5.10 (3.18–8.19)	0	0.157	7 (0.37)	3.62–8.42
Nutrition and lifestyle	Torloni MR 2009	Overweight vs. Non-overweight (cohort)	34	174233/391991	2.95 (2.68–3.24)	3.10 (2.91–3.31)	0	0.132	72 (<0.001)	1.97–4.41
Nutrition and lifestyle	Torloni MR 2009	Overweight vs. Non-overweight (case-control)	10	6214/19567	3.78 (2.49–5.76)	3.06 (2.51–3.73)	5.18 x 10^−10^	0.248	90 (<0.001)	0.83–17.2
Nutrition and lifestyle	Torloni MR 2009	Obese vs. non-obese women (cohort)	40	68013/520879	3.36 (3.01–3.74)	3.44 (3.20–3.70)	0	0.724	77 (<0.001)	1.97–5.72
Nutrition and lifestyle	Torloni MR 2009	Obese vs. non-obese women (case-control)	3	238/922	3.24 (1.28–8.19)	7.49 (4.58–12.3)	.01289	0.938	88 (0.001)	0–285401
Diseases/disorders	Pérez-López FR 2018	Endometriosis	12	1973/46789	1.14 (0.86–1.51)	0.81 (0.53–1.25)	0.3561	0.642	56 (0.009)	0.51–2.54
Diseases/disorders	Li L 2018	Obstructive sleep apnea	8	18129/56707166	1.71 (1.23–2.38)	1.89 (1.67–2.14)	1.3 x 10^−3^	0.961	83 (<0.001)	0.64–4.62
Diseases/disorders	Li L 2018	Snoring	18	2301/14216	2.14 (1.63–2.81)	6.3 (3.77–10.53)	3.54 x 10^−8^	0.015	65 (<0.001)	0.83–5.52
Diseases/disorders	Li L 2018	Sleep-disordered breathing	26	20430/56721382	1.95 (1.60–2.37)	1.89 (1.67–2.14)	2.17 x 10^−11^	0.180	72 (<0.001)	0.90–4.22
Diseases/disorders	Gong LL 2016	Overt hypothyroidism	3	3444/222161	2.44 (1.08–5.52)	1.88 (1.67–2.12)	.03262	0.688	57 (0.10)	0–15039
Diseases/disorders	Gong LL 2016	Subclinical hypothyroidism	6	1859/61708	1.59 (1.32–1.92)	1.49 (1.04–2.13)	1.29 x 10^−6^	0.208	0 (0.50)	1.22–2.07
Diseases/disorders	Gong LL 2016	Hypothyroidism (all)	7	5770/278609	1.72 (1.51–1.95)	1.88 (1.67–2.12)	4.21 x 10^−17^	0.137	14 (0.32)	1.35–2.18
Diseases/disorders	Kjerulff LE 2011	Polycystic ovary syndrome	18	2385/89669	2.83 (1.95–4.10)	2.69 (2.33–3.11)	4.63 x 10^−8^	0.653	52 (0.005)	0.94–8.46
Infections	Abariga SA 2016	Periodontitis	10	624/5100	1.66 (1.16–2.36)	1.73 (0.91–3.30)	5.18 x 10^−3^	0.008	51 (0.03)	0.61–4.49
Infections	Soepnel LM 2016	HIV infection	4	593/1070	0.83 (0.48–1.42)	1.00 (0.37–2.71)	.49148	0.472	0 (0.61)	0.25–2.71
Other	Xu Y-h 2018	Extreme sleep duration	12	2602/37140	1.43 (1.16–1.75)	1.29 (1.09–1.52)	6.8 x 10^−4^	0.039	33 (0.12)	0.88–2.32
Other	Wang JW 2018	Smoking vs non-smoking	13	22811/1341657	0.98 (0.88–1.10)	0.90 (0.81–1.00)	0.7647	0.634	49 (0.02)	0.73–1.32
Other	Wang JW 2018	Light smoking vs non-smoking	5	31257/1482334	1.10 (0.97–1.24)	1.11 (1.02–1.21)	0.1429	0.478	57 (0.05)	0.77–1.58
Other	Wang JW 2018	Heavy smoking vs non-smoking	5	17701/1064297	1.02 (0.68–1.54)	0.90 (0.81–1.00)	0.9325	0.534	60 (0.04)	0.31–3.38
Other	Xiao Y 2018	Age at menarche	5	2783/45752	1.36 (1.15–1.60)	1.34 (1.14–1.58)	3.2 x 10^−4^	0.860	33 (0.20)	0.88–2.10
Other	Moosazadeh M 2016	Family history of diabetes	33	2697/29134	3.46 (2.80–4.27)	4.36 (2.89–6.58)	5.41 x 10^−31^	0.861	76 (<0.001)	1.17–10.2
Other	Xu Y 2016	Isolated Single Umbilical Artery	7	1880/490712	1.38 (1.06–1.80)	2.08 (1.47–2.96)	.01842	0.569	35 (0.16)	0.73–2.61
Other	Pandey S 2012	IVF/ICSI versus spontaneous conception	6	13399/574391	1.31 (0.98–1.75)	1.55 (1.37–1.75)	.07039	0.169	42 (0.13)	0.63–2.72

Abbreviations: Random effects, summary odds ratio (95% CI) using random effects model; Largest effect, odds ratio (95% CI) of the largest study in the meta-analysis; Egger, p-value from Egger's regression asymmetry test for evaluation of publication bias; P, p-value; NP, not pertinent, because the estimated is larger than the observed, and there is no evidence of excess of statistical significance based on the assumption made for the plausible effect size; BMI, Body Mass Index; GDM, gestational diabetes mellitus; PA, physical activity

* Summary random effects odds ratio (95% CI) of each meta-analysis, except for three meta-analyses (Fu S 2016, Aune D 2016, Pandey S 2012 and Xiao Y 2018) where the RR was used.

‡ Odds ratio (95% CI) of the largest study in each meta-analysis, except for three meta-analyses (Fu S 2016, Aune D 2016, Pandey S 2012 and Xiao Y 2018) where the RR was used.

§ P-value from the Egger regression asymmetry test for evaluation of publication bias

|| I^2^ metric of inconsistency and P-value of the Cochran Q test for evaluation of heterogeneity

**≠** 95% Prediction Interval

Fourteen papers (47%) used the Newcastle Ottawa Scale (NOS) to qualitatively assess the included primary studies. Three papers (10%) used the Cochrane Collaboration’s risk of bias tool, three (10%) papers used the STrengthening the Reporting of OBservational studies in Epidemiology (STROBE) Statement as a quality assessment tool, and three (10%) papers used other assessment tools. Six papers (20%) did not perform any quality assessment. S1 Table summarizes these 30 papers providing data on 61 meta-analyses (comparisons), which included 697 individual study estimates.

### Quality assessment of included meta-analyses

S1 Table demonstrates the quality assessment of the included meta-analyses using the AMSTAR tool. The median AMSTAR quality score was 7.5 (IQR: 6.25–8.75). All of the meta-analyses included a comprehensive literature search and provided a comprehensive list of the characteristics of the included studies. Most of the meta-analyses did not include a list of the excluded studies while most of the meta-analyses used appropriate methods for data analysis, addressed and incorporated publication bias considerations and the authors reported the conflicts of interest.

### Summary effect sizes and significant findings

Of the 61 meta-analyses (comparisons), 51 (82%) had nominally statistically significant findings at P<0.05 using the random effects model, while only 15 (25%) remained significant after the application of the more stringent p-value threshold of P<10^−6^ (Table 1). The fifteen risk factors that presented a significant effect for an association with GDM at P<10^−6^ were the following: pre-pregnancy BMI (as a continuous variable), dietary total iron intake, low vs. normal BMI (cohort studies), overweight vs. normal BMI (cohort studies), BMI >30 vs. normal weight, BMI ~30–35 vs. normal weight, BMI >35 vs. normal weight, overweight vs. non-overweight (cohort studies), overweight vs. non-overweight (case-control), obese vs. non-obese (cohort studies), snoring, sleep-disordered breathing, hypothyroidism, polycystic ovary syndrome, and family history of diabetes. Additional information on all 61 meta-analyses is available online (S2 Table).

Across the five areas of risk factors there were differences in the proportion of associations that had nominally statistically significant summary effects. Based on the random effects calculations at P<0.05, the proportion of studies with nominally statistically significant summary effects was: 91% for biomarkers, 88% for diseases and disorders and 85% for nutrition and lifestyle. On the contrary, this was seen only in 50% of the meta-analyses on other risk factors and infections, respectively.

### Between-study heterogeneity and prediction intervals

Sixteen (26%) meta-analyses had large heterogeneity estimates (I^2^ ≥ 50% and I^2^ ≤ 75%) and 15 (25%) meta-analyses had very large heterogeneity estimates (I^2^ > 75%) (Table 1). When we calculated the 95% prediction intervals, in 18 (30%) meta-analyses the null value was excluded. This included seven biomarkers [maternal iron deficiency, ferritin levels, DQ6, thyroid antibodies (case-control studies), thyroid antibodies (all studies), 25(OH)D5 <50 nmol/l, 25(OH)D <75 nmol/l], eight nutrition and lifestyle factors [prenatal exercise (cohort studies), low vs. normal BMI (cohort studies), overweight vs. normal BMI (cohort studies), BMI >30 vs. normal weight, BMI ~30–35 vs. normal weight, BMI >35 vs. normal weight, overweight vs. non-overweight (cohort studies), obese vs. non-obese (cohort studies)], two diseases and disorders (subclinical hypothyroidism and hypothyroidism), and one other risk factor (family history of diabetes) (Table 1).

### Small-study effects and excess significance bias

Evidence for statistically significant small-study effects (Egger test P<0.10 and random effects summary estimate larger compared to the point estimate of the largest study in the meta-analysis) was identified in 5 out of 61 (8%) meta-analyses (S2 Table, available online). These included four meta-analyses on biomarkers (hemoglobin concentration, mean platelet volume, serum retinol-binding protein-4, DQ2), and one on other factors (Extreme sleep duration). Eight (13%) associations had hints of excess statistical significance bias with statistically significant (P<0.05) excess of positive studies under any of the three assumptions for the plausible effect size—the fixed effects summary, the random effects summary or the results of the largest study (S2 Table). Four (50%) of them pertained to biomarkers, three (38%) pertained to nutrition and lifestyle, and one (12%) pertained to other risk factors. Table 2 shows the results of excess of statistical significance bias according to category of risk factor.

**Table 2:** Observed and expected number of positive studies by type of risk factor*.

Area	No. of studies	Observed positive	Expected positive (fixed) †	P‡ (fixed)	Expected positive (random)§	P‡ (random)	Expected positive (largest)‖	P‡ (largest)	Expected positive (composite) ¶	P‡ (composite)
All	697	365	419	0.00	447	0.00	410	0.00	410	0.00
Biomarkers	270	117	113	0.67	137	0.02	106	0.17	110	0.15
Nutrition and lifestyle	229	160	181	0.00	182	0.00	181	0.00	181	0.00
Diseases & disorders	95	41	64	0.00	69	0.00	73	0.00	63	0.00
Infections	15	3	4	0.77	6	0.28	5	0.28	4	0.77
Other	86	44	57	0.01	54	0.03	44	0.91	44	0.91

* NP, not pertinent, because the estimated is larger than the observed, and there is no evidence of excess of statistical significance based on the assumption made for the plausible effect size.

† Expected number of statistically significant studies using the summary fixed effects estimate of each meta-analysis as the plausible effect size.

‡ P value of the excess of statistically significant test. All statistical tests were two-sided.

§ Expected number of statistically significant studies using the summary random effects estimate of each meta-analysis as the plausible effect size.

‖ Expected number of statistically significant studies using the effect of the largest study of each meta-analysis as the plausible effect size.

¶ Expected number of statistically significant studies using the most conservative of the three estimates (fixed effects summary, random effects summary, largest study) of each meta-analysis as the plausible effect size.

### Risk factors with strong evidence of association

After applying our credibility criteria, four risk factors, low vs. normal BMI (cohort studies), BMI ~30–35 vs. normal weight, BMI >35 vs. normal weight, and hypothyroidism (all types) presented convincing evidence for an association with GDM, supported by more than 1000 cases, P<10^−6^ under the random effect model, no hints for small-study effects and for excess statistical significance, not large heterogeneity (I^2^<50%), and a 95% PI excluding the null value. Ten risk factors [pre-pregnancy BMI (as a continuous variable), overweight vs. normal BMI (cohort), BMI >30 vs. normal weight, overweight vs. non-overweight (cohort), overweight vs. non-overweight (case-control), obese vs. non-obese (cohort), snoring, sleep-disordered breathing, polycystic ovary syndrome, family history of diabetes] presented highly suggestive evidence for GDM.

Nine risk factors were supported by suggestive evidence and twenty-seven associations presented weak evidence (P<0.05). An overall assessment of statistically significant associations for GDM is presented in Table 3.

**Table 3:** Assessment across the statistically significant associations for gestational diabetes.

Level of evidence	Criteria used	Decreased risk	Increased risk
**Convincing**	>1000 cases, ^a^ P<10^−6^, not large heterogeneity (I^2^ <50%), 95% prediction interval excluding the null value, no evidence for small-study effects ^b^ and excess significance bias ^c^	Low vs. Normal BMI (cohort)	BMI ~30–35 vs. Normal weight, BMI >35 vs. Normal weight, Hypothyroidism (all)
**Highly suggestive**	>1000 cases, ^a^ P<10^−6^ and nominally statistically significant effect present at the largest study		Pre-pregnancy BMI (as a continuous variable), Overweight vs. Normal BMI (cohort), BMI >30 vs. normal weight, Overweight vs. Non-overweight women (cohort), Overweight vs. Non-overweight (case-control), Obese vs. non-obese women (cohort), Snoring, Sleep-disordered breathing, Polycystic ovary syndrome, Family history of diabetes
**Suggestive**	>1000 cases, ^a^ P<10^−3^	Low vs. Normal BMI (case-control), Serum 25(OH)D level	Hemoglobin concentration (g/dL), Mean platelet volume, Insufficient vitamin D, Dietary total iron intake, Subclinical hypothyroidism, Extreme sleep duration, Age at menarche
**Weak**	The rest associations with ^a^ P < 0.05	Maternal iron deficiency, DQ6, Selenium level, Prenatal exercise + co-interventions, Prenatal exercise (cohort studies), Prenatal exercise (cross-sectional studies)	Chemerin levels, Ferritin concentration (ng/mL), Betatrophin levels, Ferritin (highest vs lowest ferritin levels) (cohorts), Serum ferritin (GMT-women vs non-GMD), Hemoglobin levels, Ferritin (highest vs lowest ferritin levels) (mixed), Serum retinol-binding protein-4, DQ2, DR13, DR17, Thyroid antibodies (case-control), Thyroid antibodies (All studies), 25(OH)D5 <50 nmol/l, 25(OH)D <75 nmol/l, Overweight vs. Normal BMI (case-control), Obese vs. non-obese women (case-control), Obstructive sleep apnea, Overt hypothyroidism, Periodontitis, Isolated Single Umbilical Artery

Abbreviations: BMI, Body Mass Index; GDM, gestational diabetes mellitus.

^a^ P indicates the P-values of the meta-analysis random effects model.

^b^ Small study effect is based on the P-value from the Egger’s regression asymmetry test (P<0.10).

^c^ Based on the P-value (P<0.05) of the excess significance test using the largest study (smallest standard error) in a meta-analysis as the plausible effect size.

## Discussion

### Main findings

In this umbrella review we evaluated the current evidence, derived from meta-analyses of observational studies on the association between various risk factors and GDM. Overall, from the 61 associations that have been examined, only a minority had strongly significant results with no suggestion of bias, as can be inferred by substantial heterogeneity between studies, small study effects, and excess significance bias. Four risk factors were supported by convincing evidence, including low vs. normal BMI (cohort studies), BMI ~30–35 vs. normal weight, BMI >35 vs. normal weight, and hypothyroidism. Another ten risk factors from various fields [pre-pregnancy BMI (as a continuous variable), overweight vs. normal BMI (cohort), BMI >30 vs. normal weight, overweight vs. non-overweight (cohort), overweight vs. non-overweight (case-control), obese vs. non-obese (cohort), snoring, sleep-disordered breathing, polycystic ovary syndrome, family history of diabetes], achieved highly suggestive evidence for an association with GDM.

### Interpretation in light of evidence

It is well-known that maternal weight, as determined from pre-conception BMI, is critical on the development of insulin resistance and type II diabetes as well as GDM. This summary of observational studies shows that the more robust associations were related to overweight and obesity, as three out of four associations that met the criteria for convincing evidence and six out of ten highly suggestive associations were concentrated on maternal pre-pregnancy BMI and the risk of GDM. The association of low BMI vs. normal BMI was the only protective factor, which it was supported by convincing evidence for protection against GDM.

Our findings further support the current guidelines regarding pregnancy weight, nutrition and activity, issued from the National Institute for Health and Clinical Excellence (NICE), the Institute of Medicine (IOM) and the American College of Obstetricians and Gynecologists (ACOG), which they accepted lifestyle change as an essential component of prevention and management of GDM.[77–79] NICE recommendations include specific guidelines for healthy eating, low-fat diet and moderate physical activity before, during, and after pregnancy.[78] Preventive measures against gestational diabetes may include diet and exercise as described on the most recent Cochrane review of interventions from moderate quality evidence. Nevertheless, the variability of the diet and exercise components tested in the included studies, make the evidence insufficient to inform practice.[80] Large, well-designed, RCTs are needed to confirm the effectiveness of pre-conception weight and gestational weight gain reduction and the effects of dietary interventions in pregnancy for preventing GDM in different categories of pre-pregnancy BMI with special focus on overweight and obese women.

The observed association between obesity and GDM is biologically plausible. Normal pregnancy is characterized by a state of insulin resistance defined as an impaired response to insulin. This physiological insulin resistance also occurs in women with GDM on a background of chronic insulin resistance due to obesity to which the insulin resistance of pregnancy is partially additive. Obesity can cause major changes in maternal intermediary metabolism, where co-existing conditions associated with increased insulin resistance, higher serum lipids, and lower plasma levels of adiponectin, appear to play a central role to the development of GDM.[81–83]

The association between hypothyroidism, which includes both subclinical and overt hypothyroidism, and risk of GDM, was supported by convincing evidence. Increased levels of human chorionic gonadotropin (hCG) in the first trimester of pregnancy directly stimulate the thyroid gland to increase production of thyroid hormone, which leads in decreased secretion of thyroid stimulating hormone (TSH).[84] Proposed mechanisms that describe the relationship between hypothyroidism and gestational diabetes are supported from studies that show that both overt and subclinical hypothyroidism can lead to significantly increased insulin resistance.[85–88] Although, these findings would suggest that routine screening of thyroid hormones during pregnancy could be essential, universal thyroid screening in pregnancy is controversial.[89] The most recent ACOG recommendations suggest testing only women at high risk of thyroid disease before they become pregnant or when they are early in pregnancy.[90] On the contrary, the American Thyroid Association [91] and the Endocrine Society [92] call for universal thyroid-function screening early in pregnancy. On the side of this controversy, women with known thyroid disease could be offered GDM screening earlier in pregnancy.

In the current umbrella review, we applied a transparent and replicable set of criteria and statistical tests to evaluate and categorize the level of existing observational evidence within in five broad areas with the goal to detect biases that work on a field-wide level. Although, 82% of the included meta-analyses report a nominally (P<0.05) statistically significant random-effects summary estimate, when stringent P value was considered (P<10^−6^), the proportion of significant associations decreased to 25%. Thirty-one (51%) associations had large or very large heterogeneity, while when we calculated the 95% prediction intervals, which further account for heterogeneity, we found that the null value was excluded in more than half of the associations. Only four of the assessed risk factors found to provide convincing evidence, indicating that several published meta-analyses of observational studies in the field could be susceptible to biases and the reported associations in the existing studies are often exaggerated.

The ability to modify those factors, mainly those related to overweight and obesity, through clinical interventions or public health policy measures remains to be established. Furthermore, there is no guarantee that even a convincing observational association for a modifiable risk factor would necessarily translate into large preventive benefits for GDM if these risk factors were to be modified.[93] With obesity becoming a global epidemic, the assessment of the strength of the evidence supporting the impact of overweight and obesity in GDM could allow the identification of women at high risk for adverse outcomes and allow better prevention. Obesity is generating an unfavorable metabolic environment from early gestation; therefore, initiation of interventions for weight loss during pregnancy might be belated to prevent or reverse adverse effects, which highlights the need of weight management strategies before conception.[94] GDM does not only increase the risk for maternal and fetal complication in pregnancy, but also significantly increases a woman’s risk of type 2 diabetes, metabolic syndrome (characterized by glucose intolerance, central obesity, dyslipidemia, and insulin resistance), and cardiovascular disease (CVD) after pregnancy.[95–98]

### Limitations

Umbrella reviews focus on existing systematic reviews and meta-analyses and therefore some studies may have not been included either because the original systematic reviews did not identify them, or they were too recent to be included. In the current assessment we used all available data from observational studies, therefore the meta-analysis estimates may partly reflect the biases from which the original studies suffer from. Statistical tests of bias in the body of evidence (small study effect and excess significance tests) offer hints of bias, not definitive proof thereof, while the Egger test is difficult to interpret when the between-study heterogeneity is large. These tests have low power if the meta-analyses include less than 10 studies and they may not identify the exact source of bias.[39, 41, 99] Furthermore, we did not appraise the quality of the individual studies on our own, since this should be included in the original meta-analysis and it was beyond the scope of the current umbrella review. However, we recorded whether and how they performed a quality assessment of the synthesized studies. Lastly, we cannot exclude the possibility of selective reporting for some associations in several studies. For example, perhaps some risk factors were more likely to be reported, if they had statistically significant results.

## Conclusion

The present umbrella review of meta-analyses identified 61 unique risk factors for GDM. Our analysis identified four risk factors with convincing evidence and strong epidemiological credibility pertaining to hypothyroidism and BMI (specifically, low vs. normal BMI (cohort studies), BMI ~30–35 vs. normal weight, BMI >35 vs. normal weight). Diet and lifestyle modifications in pregnancy should be tested in large randomized trials. Our findings suggest that women with known thyroid disease could be offered screening for GDM earlier in pregnancy. As previously suggested, the use of standardized definitions and protocols for exposures, outcomes, and statistical analyses may diminish the threat of biases, allow for the computation of more precise estimates and will promote the development and training of prediction models that could promote public health.

## Supporting information

S1 Table AMSTAR tool for evaluation of quality of included meta-analyses. Note: Y: Yes, N: No, CA: Cannot Answer. Item 1: Was an ‘‘a priori” design provided? Item 2: Was there duplicate study selection and data extraction? Item 3: Was a comprehensive literature search performed? Item 4: Was the status of publication (i.e., grey literature) used as an inclusion criterion? Item 5: Was a list of studies (included and excluded) provided? Item 6: Were the characteristics of the included studies provided? Item 7: Was the scientific quality of the included studies assessed and documented? Item 8: Was the scientific quality of the included studies used appropriately in formulating conclusions? Item 9: Were the methods used to combine the findings of studies appropriate? Item 10: Was the likelihood of publication bias assessed? Item 11: Was the conflict of interest included?
(DOCX)

S2 Table Analytical description of the 62 selected meta-analyses with observed and expected number of "positive" study datasets. Abbreviations: Random effects, summary odds ratio (95% CI) using random effects model; Largest effect, odds ratio (95% CI) of the largest study in the meta-analysis; Egger, p-value from Egger's regression asymmetry test for evaluation of publication bias; P, p-value; NP, not pertinent, because the estimated is larger than the observed, and there is no evidence of excess of statistical significance based on the assumption made for the plausible effect size; BMI, Body Mass Index; GDM, gestational diabetes mellitus; PA, physical activity.* Summary random effects odds ratio (95% CI) of each meta-analysis, except for three meta-analyses (Fu S 2016, Aune D 2016, Pandey S 2012 and Xiao Y 2018) where the RR was used. † Summary fixed effects odds ratio (95% CI) of each meta-analysis, except for three meta-analyses (Fu S 2016, Aune D 2016, Pandey S 2012 and Xiao Y 2018) where the RR was used.‡ Odds ratio (95% CI) of the largest study in each meta-analysis, except for three meta-analyses (Fu S 2016, Aune D 2016, Pandey S 2012 and Xiao Y 2018) where the RR was used.§ P-value from the Egger regression asymmetry test for evaluation of publication bias|| I2 metric of inconsistency (95% confidence intervals of I2) and P-value of the Cochran Q test for evaluation of heterogeneity.
≠ 95% Prediction Interval ¶ Observed number of statistically significant studies # Expected number of statistically significant studies using the summary fixed effects estimate of each meta-analysis as the plausible effect size** P-value of the excess statistical significance test.
¥ Expected number of statistically significant studies using the summary random effects estimate of each meta-analysis as the plausible effect size ȣ Expected number of statistically significant studies using the effect of the largest study of each meta-analysis as the plausible effect size.
(DOCX)
