# Supplementary material for: Risk factors for gestational diabetes: An umbrella review of meta-analyses of observational studies
Source: PLoS One. 2019 Apr 19;14(4):e0215372. doi: 10.1371/journal.pone.0215372 (PMC6474596; doi:10.1371/journal.pone.0215372)
Supplement: S1 Table — Note: Y: Yes, N: No, CA: Cannot Answer. Item 1: Was an ‘‘a priori” design provided? Item 2: Was there duplicate study selection and data extraction? Item 3: Was a comprehensive literature search performed? Item 4: Was the status of publication (i.e., grey literature) used as an inclusion criterion? Item 5: Was a list of studies (included and excluded) provided? Item 6: Were the characteristics of the included studies provided? Item 7: Was the scientific quality of the included studies assessed and documented? Item 8: Was the scientific quality of the included studies used appropriately in formulating conclusions? Item 9: Were the methods used to combine the findings of studies appropriate? Item 10: Was the likelihood of publication bias assessed? Item 11: Was the conflict of interest included? (DOCX) [file pone.0215372.s001.docx]

| **Reference** | **AMSTAR Item** | | | | | | | | | | | **Total** |
| --- | --- | --- | --- | --- | --- | --- | --- | --- | --- | --- | --- | --- |
|  | **1** | **2** | **3** | **4** | **5** | **6** | **7** | **8** | **9** | **10** | **11** |  |
| Xiao Y, 2018 ^(57)^ | CA | CA | Y | N | N | Y | Y | Y | Y | N | Y | **6** |
| Wang JW, 2018 ^(69)^ | CA | CA | Y | N | N | Y | Y | N | Y | Y | Y | **6** |
| Zhou Z, 2018 ^(70)^ | CA | Y | Y | CA | N | Y | Y | CA | Y | Y | Y | **7** |
| Zhang W, 2018 ^(71)^ | Y | Y | Y | Y | N | Y | Y | CA | Y | Y | Y | **7** |
| Perez Lopez FR, 2017 ^(72)^ | CA | Y | Y | N | N | Y | N | Y | Y | Y | Y | **7** |
| Tiongco RE, 2018 ^(66)^ | CA | CA | Y | CA | N | Y | Y | N | Y | Y | Y | **6** |
| Zhou Z, 2018 ^(60)^ | CA | Y | Y | CA | N | Y | Y | Y | Y | Y | Y | **8** |
| Kataria Y, 2018 ^(74)^ | N | CA | N | CA | N | Y | Y | CA | Y | Y | Y | **5** |
| Davenport, 2018 ^(17)^ | Y | Y | Y | Y | Y | Y | Y | Y | Y | Y | Y | **11** |
| Najafi F, 2018 ^(50)^ | CA | Y | Y | CA | N | Y | Y | Y | Y | Y | Y | **8** |
| Xu YH, 2018 ^(51)^ | N | Y | Y | N | N | Y | Y | N | Y | Y | Y | **7** |
| Li L, 2018 ^(52)^ | N | Y | Y | N | N | Y | N | N | Y | Y | Y | **9** |
| Amraei M, 2018 ^(67)^ | CA | Y | Y | N | N | Y | N | N | Y | Y | Y | **7** |
| Abariga SA, 2016 ^(46)^ | CA | Y | Y | CA | N | Y | Y | Y | Y | Y | Y | **8** |
| Fu S, 2016 ^(47)^ | CA | Y | Y | Y | N | Y | Y | Y | Y | Y | Y | **9** |
| Aune D, 2016 ^(68)^ | CA | Y | Y | Y | N | Y | Y | Y | Y | Y | Y | **9** |
| Fernandez-Cao JC, 2016 ^(73)^ | Y | Y | Y | Y | N | Y | Y | Y | Y | Y | Y | **10** |
| Gong LL, 2016 ^(59)^ | CA | CA | Y | CA | N | Y | N | N | Y | Y | Y | **5** |
| Guo C, 2016 ^(64)^ | CA | Y | Y | CA | N | Y | Y | Y | Y | Y | Y | **8** |
| Hu S, 2016 ^(65)^ | N | CA | Y | N | N | Y | Y | Y | Y | Y | Y | **7** |
| Kjerulff LE, 2011 ^(63)^ | CA | Y | Y | CA | N | Y | CA | CA | CA | CA | Y | **4** |
| Kong FJ, 2016 ^(62)^ | CA | Y | Y | CA | N | Y | Y | Y | Y | Y | Y | **8** |
| Kong FJ, 2017 ^(61)^ | CA | Y | Y | CA | N | Y | Y | Y | Y | Y | Y | **8** |
| Moosazadeh M, 2017 ^(48)^ | CA | Y | Y | CA | N | Y | Y | CA | Y | Y | CA | **6** |
| Padley S, 2012 ^(49)^ | CA | Y | Y | Y | CA | Y | Y | Y | Y | Y | Y | **9** |
| Torloni MR, 2009 ^(54)^ | CA | Y | Y | Y | CA | Y | Y | Y | Y | Y | Y | **9** |
| Soepnel LM, 2016 ^(53)^ | Y | Y | Y | CA | N | Y | Y | Y | Y | Y | Y | **9** |
| Wei SQ, 2013 ^(55)^ | CA | Y | Y | CA | N | Y | Y | Y | Y | Y | Y | **8** |
| Xu Υ, 2016 ^(56)^ | CA | CA | Y | CA | CA | Y | CA | CA | Y | Y | Y | **5** |
| Yang Υ, 2015 ^(58)^ | CA | Y | Y | CA | N | Y | Y | CA | Y | Y | Y | **7** |

S1 Table: AMSTAR tool for evaluation of quality of included meta-analyses

Y: Yes, N: No, CA: Cannot Answer. Item 1: Was an ‘‘a priori’’ design provided?, Item 2: Was there duplicate study selection and data extraction? Item 3: Was a comprehensive literature search performed? Item 4: Was the status of publication (i.e., grey literature) used as an inclusion criterion? Item 5: Was a list of studies (included and excluded) provided? Item 6: Were the characteristics of the included studies provided? Item 7: Was the scientific quality of the included studies assessed and documented? Item 8: Was the scientific quality of the included studies used appropriately in formulating conclusions? Item 9: Were the methods used to combine the find- ings of studies appropriate? Item 10: Was the likelihood of publication bias assessed? Item 11: Was the conflict of interest included?
