# Supplementary material for: Risk factors for gestational diabetes: An umbrella review of meta-analyses of observational studies
Source: PLoS One. 2019 Apr 19;14(4):e0215372. doi: 10.1371/journal.pone.0215372 (PMC6474596; doi:10.1371/journal.pone.0215372)
Supplement: S2 Table — Abbreviations: Random effects, summary odds ratio (95% CI) using random effects model; Largest effect, odds ratio (95% CI) of the largest study in the meta-analysis; Egger, p-value from Egger's regression asymmetry test for evaluation of publication bias; P, p-value; NP, not pertinent, because the estimated is larger than the observed, and there is no evidence of excess of statistical significance based on the assumption made for the plausible effect size; BMI, Body Mass Index; GDM, gestational diabetes mellitus; PA, physical activity.* Summary random effects odds ratio (95% CI) of each meta-analysis, except for three meta-analyses (Fu S 2016, Aune D 2016, Pandey S 2012 and Xiao Y 2018) where the RR was used. † Summary fixed effects odds ratio (95% CI) of each meta-analysis, except for three meta-analyses (Fu S 2016, Aune D 2016, Pandey S 2012 and Xiao Y 2018) where the RR was used.‡ Odds ratio (95% CI) of the largest study in each meta-analysis, except for three meta-analyses (Fu S 2016, Aune D 2016, Pandey S 2012 and Xiao Y 2018) where the RR was used.§ P-value from the Egger regression asymmetry test for evaluation of publication bias|| I2 metric of inconsistency (95% confidence intervals of I2) and P-value of the Cochran Q test for evaluation of heterogeneity. ≠ 95% Prediction Interval ¶ Observed number of statistically significant studies # Expected number of statistically significant studies using the summary fixed effects estimate of each meta-analysis as the plausible effect size** P-value of the excess statistical significance test. ¥ Expected number of statistically significant studies using the summary random effects estimate of each meta-analysis as the plausible effect size ȣ Expected number of statistically significant studies using the effect of the largest study of each meta-analysis as the plausible effect size. (DOCX) [file pone.0215372.s002.docx]

| **S2 Table. Analytical description of the 62 selected meta-analyses with observed and expected number of "positive" study datasets** | | | | | | | | | | | | | | |  |  | | | |  |  |  | |  | |  | |  |  | |
| --- | --- | --- | --- | --- | --- | --- | --- | --- | --- | --- | --- | --- | --- | --- | --- | --- | --- | --- | --- | --- | --- | --- | --- | --- | --- | --- | --- | --- | --- | --- |
| **Area** | **Author, year** | **Comparison** | | | **Studies** | | **Cases/controls** | | **Random effects*** | | **Fixed effects†** | | **Largest effect‡** | | **Egger§** | **I^2^ (95% CI) (P)\|\|** | | **95% PI ≠** | | **O¶** | **E # (fixed)** | **P** (fixed)** | | **E** ¥ **(random)** | | **P** (random)** | | **E** ȣ  **(largest)** | **P****  **(largest)** | |
| Biomarkers | Zhou Z 2018 | Chemerin levels | | | 13 | | 742/840 | | 5.40 (1.28-22.8) | | 6.05 (4.87-7.53) | | 127.8 (82-199.1) | | 0.591 | 97 (97-98) (<0.001) | | 0.02-1863 | | 7 | 12.4 | NP | | 12.10 | | NP | | 13.0 | NP | |
| Biomarkers | Zhang W 2018 | Visfatin levels | | | 26 | | 1033/1272 | | 1.57 (0.86-2.83) | | 1.69 (1.44-1.98) | | 2.34 (1.54-3.55) | | 0.764 | 92 (90-94) (<0.001) | | 0.07-33.76 | | 16 | 6.58 | 0.00 | | 5.20 | | 0.00 | | 13.0 | 0.33 | |
| Biomarkers | Kataria Y 2018 | Ferritin concentration (ng/mL) | | | 12 | | 2152/46443 | | 16.38 (2.77-96.9) | | 2.83 (2.49-3.20) | | 1.36 (1.05-1.77) | | 0.108 | 99 (NA) (<0.001) | | 0.01-23068 | | 10 | 10.6 | 0.64 | | 12.0 | | NP | | 3.78 | NP | |
| Biomarkers | Kataria Y 2018 | Hemoglobin concentration (g/dL) | | | 9 | | 1022/3531 | | 4.34 (2.07-9.08) | | 1.35 (1.19-1.55) | | 1.39 (1.05-1.83) | | 0.013 | 96 (95-97) (<0.001) | | 0.30-62.4 | | 5 | 2.75 | 0.14 | | 8.78 | | NP | | 3.02 | 0.17 | |
| Biomarkers | Zhou Z 2018 | Mean platelet volume | | | 20 | | 1466/1951 | | 4.09 (2.24-7.47) | | 2.05 (1.82-2.32) | | 0.67 (0.51-0.89) | | 0.017 | 95 (94-96) (<0.001) | | 0.24-69.6 | | 14 | 11.5 | 0.27 | | 18.9 | | NP | | 5.06 | 0.00 | |
| Biomarkers | Amraei M 2018 | Insufficient vitamin D | | | 26 | | 5464/15039 | | 1.39 (1.18-1.63) | | 1.31 (1.17-1.46) | | 1.58 (0.85-3.78) | | 0.068 | 43 (0-63) (0.011) | | 0.80-2.41 | | 7 | 6.11 | 0.65 | | 8.10 | | 0.83 | | 12.6 | 0.03 | |
| Biomarkers | Amraei M 2018 | Serum 25(OH)D level | | | 16 | | 1337/4158 | | 0.62 (0.49-0.78) | | 0.63 (0.55-0.71) | | 0.56 (0.34-0.93) | | 0.681 | 69 (43-80) (<0.001) | | 0.26-1.46 | | 11 | 6.92 | 0.05 | | 7.16 | | 0.08 | | 9.25 | 0.45 | |
| Biomarkers | Tiongco RE 2018 | Maternal iron deficiency | | | 6 | | 358/14799 | | 0.61 (0.47-0.80) | | 0.61 (0.47-0.80) | | 0.80 (0.32-1.99) | | 0.864 | 0 (0-61) (0.687) | | 0.42-0.89 | | 2 | 3.02 | 0.45 | | 3.02 | | 0.45 | | 0.91 | 0.23 | |
| Biomarkers | Kong FJ 2017 | Betatrophin levels | | | 8 | | 401/421 | | 6.65 (2.12-20.9) | | 10.5 (7.92-13.9) | | 16.5 (9.18-29.8) | | 0.191 | 94 (90-95) (<0.001) | | 0.11-411.7 | | 8 | 7.97 | 1.00 | | 7.82 | | 1.00 | | 8.00 | 1.00 | |
| Biomarkers | Fu S 2016 | Ferritin (highest vs lowest) (cohorts) | | | 4 | | 214/1662 | | 3.22 (1.73-6.00) | | 3.22 (1.73-6.00) | | 4.98 (1.46-17.03) | | 0.953 | 0 (0-68) (0.815) | | 0.82-12.65 | | 2 | 2.76 | 0.59 | | 2.76 | | 0.59 | | 3.40 | 0.11 | |
| Biomarkers | Fu S 2016 | Serum ferritin (GMD-women vs non-GMD) | | | 6 | | 403/498 | | 4.89 (2.06-11.58) | | 4.51 (3.50-5.82) | | 6.45 (4.07-10.24) | | 0.756 | 91 (83-94) (<0.001) | | 0.22-106.6 | | 5 | 5.90 | 0.10 | | 5.93 | | 0.07 | | 5.99 | 0.01 | |
| Biomarkers | Fernández-Cao JC 2016 | Hemoglobin levels (highest vs lowest levels) | | | 9 | | 792/4393 | | 1.54 (1.18-2.03) | | 1.53 (1.24-1.89) | | 0.81 (0.36-1.82) | | 0.752 | 33 (0-68) (0.157) | | 0.81-2.93 | | 3 | 3.58 | 1.00 | | 3.72 | | 0.74 | | 1.24 | 0.12 | |
| Biomarkers | Fernández-Cao JC 2016 | Ferritin levels (highest vs lowest levels) | | | 7 | | 330/5574 | | 2.09 (1.48-2.96) | | 2.09 (1.48-2.96) | | 2.27 (1.20-4.30) | | 0.600 | 1 (0-68) (0.42) | | 1.31-3.34 | | 3 | 5.40 | 0.05 | | 5.40 | | 0.05 | | 5.79 | 0.02 | |
| Biomarkers | Hu S 2016 | Serum retinol-binding protein-4 | | | 17 | | 647/620 | | 4.38 (2.10-9.14) | | 2.64 (2.12-3.27) | | 1.27 (0.70-2.30) | | 0.025 | 91 (88-93) (<0.001) | | 0.18-106.7 | | 9 | 9.77 | 0.81 | | 14.2 | | NP | | 1.45 | 0.00 | |
| Biomarkers | Iliodromiti S 2016 | Adiponectin | | | 11 | | 794/2071 | | 6.35 (4.08-9.88) | | 6.27 (5.09-7.72) | | 5.05 (3.55-7.18) | | 0.770 | 71 (37-83) (<0.001) | | 1.56-25.9 | | 10 | 10.6 | 0.37 | | 10.6 | | 0.36 | | 10.3 | 0.52 | |
| Biomarkers | Guo CC 2016 | DQ2 | | | 12 | | 2333/2687 | | 1.36 (1.10-1.66) | | 1.20 (1.06-1.36) | | 0.96 (0.79-1.16) | | 0.008 | 43 (0-69) (0.06) | | 0.80-2.30 | | 4 | 2.03 | 0.13 | | 3.91 | | 1.00 | | 0.68 | 0.00 | |
| Biomarkers | Guo CC 2016 | DQ6 | | | 11 | | 2270/2576 | | 0.81 (0.69-0.94) | | 0.81 (0.69-0.94) | | 0.75 (0.55-1.02) | | 0.551 | 0 (0-51) (0.743) | | 0.67-0.97 | | 1 | 2.40 | 0.48 | | 2.40 | | 0.48 | | 3.52 | 0.19 | |
| Biomarkers | Guo CC 2016 | DR 13 | | | 4 | | 209/225 | | 2.46 (1.02-5.90) | | 2.54 (1.62-3.99) | | 0.73 (0.29-1.87) | | 0.982 | 67 (0-87) (0.03) | | 0.07-88.5 | | 2 | 2.82 | 0.59 | | 2.71 | | 0.60 | | 0.57 | 0.10 | |
| Biomarkers | Guo CC 2016 | DR17 | | | 5 | | 329/335 | | 3.16 (1.31-7.64) | | 2.59 (1.61-4.18) | | 3.13 (1.11-8.81) | | 0.116 | 69 (0-86) (0.01) | | 0.16-62.9 | | 3 | 4.01 | 0.26 | | 4.54 | | 0.07 | | 4.51 | 0.08 | |
| Biomarkers | Yang Y 2015 | Thyroid antibodies (cohort) | | | 11 | | 1596/30012 | | 1.07 (0.97-1.19) | | 1.07 (0.97-1.19) | | 1.18 (0.77-1.81) | | 0.546 | 0 (0-51) (0.44) | | 0.95-1.21 | | 1 | 0.78 | 0.56 | | 0.78 | | 0.56 | | 1.87 | 0.70 | |
| Biomarkers | Yang Y 2015 | Thyroid antibodies (case-control) | | | 10 | | 856/2062 | | 1.21 (1.05-1.41) | | 1.21 (1.05-1.41) | | 1.33 (1.09-1.63) | | 0.402 | 0 (0-53) (0.73) | | 1.02-1.44 | | 1 | 1.25 | 1.00 | | 1.25 | | 1.00 | | 1.90 | 0.70 | |
| Biomarkers | Yang Y 2015 | Thyroid antibodies (All studies) | | | 21 | | 2452/32074 | | 1.12 (1.03-1.22) | | 1.12 (1.03-1.22) | | 1.18 (0.77-1.81) | | 0.485 | 0 (0-41) (0.60) | | 1.02-1.23 | | 2 | 1.91 | 1.00 | | 1.91 | | 1.00 | | 2.93 | 0.76 | |
| Biomarkers | Wei SQ 2013 | 25(OH)D5<50 nmol/l | | | 10 | | 623/3503 | | 1.37 (1.11-1.70) | | 1.37 (1.11-1.70) | | 1.20 (0.72-2.00) | | 0.147 | 0 (0-53) (0.51) | | 1.07-1.76 | | 1 | 2.24 | 0.70 | | 2.24 | | 0.70 | | 1.06 | 1.00 | |
| Biomarkers | Wei SQ 2013 | 25(OH)D<75 nmol/l | | | 8 | | 542/3298 | | 1.52 (1.17-1.98) | | 1.53 (1.19-1.96) | | 1.63 (0.79-3.33) | | 0.954 | 7 (0-59) (0.37) | | 1.01-2.30 | | 2 | 3.09 | 0.72 | | 3.06 | | 0.72 | | 3.85 | 0.29 | |
|  |  |  | | |  | |  | |  | |  | |  | |  |  | |  | |  |  |  | |  | |  | |  |  | |
| Nutrition and lifestyle | Najafi F 2018 | Pre-pregnancy BMI (as a continuous variable) | | | 5 | | 1605/3112 | | 1.19 (1.13-1.26) | | 1.16 (1.15-1.18) | | 1.16 (1.14-1.18) | | 0.499 | 82 (48-91) (<0.001) | | 0.98-1.44 | | 5 | 1.23 | 0.00 | | 1.40 | | 0.00 | | 1.21 | 0.00 | |
| Nutrition and lifestyle | Davenport 2018 | Prenatal exercise + co-interventions | | | 4 | | 81/265 | | 0.47 (0.25-0.89) | | 0.46 (0.26-0.78) | | 0.41 (0.20-0.86) | | 0.719 | 16 (0-73) (0.31) | | 0.08-2.92 | | 2 | 1.40 | 0.62 | | 1.33 | | 0.60 | | 1.67 | 1.00 | |
| Nutrition and lifestyle | Davenport 2018 | Prenatal exercise (cohort studies) | | | 14 | | 343/9252 | | 0.68 (0.53-0.87) | | 0.68 (0.53-0.87) | | 0.59 (0.43-0.82) | | 0.259 | 0 (0-47) (0.62) | | 0.52-0.89 | | 1 | 2.28 | 0.71 | | 2.28 | | 0.71 | | 3.16 | 0.22 | |
| Nutrition and lifestyle | Davenport 2018 | Prenatal exercise (cross-sectional studies) | | | 8 | | 136/5504 | | 0.66 (0.44-0.97) | | 0.66 (0.44-0.97) | | 0.63 (0.32-1.26) | | 0.232 | 0 (0-56) (0.69) | | 0.40-1.07 | | 0 | 1.24 | 0.62 | | 1.24 | | 0.62 | | 1.41 | 0.37 | |
| Nutrition and lifestyle | Davenport 2018 | Prenatal exercise (case-control studies) | | | 4 | | 196/451 | | 0.54 (0.23-1.27) | | 0.72 (0.49-1.06) | | 0.99 (0.60-1.62) | | 0.367 | 62 (0-85) (0.05) | | 0.02-13.6 | | 2 | 0.73 | 0.15 | | 1.84 | | 1.00 | | 0.20 | 0.01 | |
| Nutrition and lifestyle | Fu S 2016 | Dietary total iron intake | | | 3 | | 1007/13850 | | 1.01 (1.00-1.01) | | 1.01 (1.00-1.01) | | 1.12 (0.87-1.45) | | NA | 0 (0-73) (0.73) | | 0.99-1.03 | | 2 | 0.15 | 0.01 | | 0.15 | | 0.01 | | 0.59 | 0.10 | |
| Nutrition and lifestyle | Kong FJ 2016 | Selenium level | | | 7 | | 178/391 | | 0.12 (0.03-0.53) | | 0.21 (0.14-0.30) | | 0.12 (0.06-0.26) | | 0.499 | 93 (89-95) (<0.001) | | 0.00-19.81 | | 6 | 5.73 | 1.00 | | 6.52 | | 0.39 | | 6.51 | 0.40 | |
| Nutrition and lifestyle | Aune D 2016 | Leisure-time PA before pregnancy | | | 8 | | 2401/30191 | | 0.78 (0.61-1.00) | | 0.78 (0.67-0.90) | | 0.81 (0.68-1.01) | | 0.869 | 47 (0-75) (0.07) | | 0.41-1.47 | | 4 | 3.28 | 0.72 | | 3.25 | | 0.72 | | 2.70 | 0.46 | |
| Nutrition and lifestyle | Aune D 2016 | Leisure-time PA during pregnancy | | | 5 | | 580/5140 | | 0.97 (0.73-1.28) | | 0.97 (0.73-1.28) | | 0.91 (0.37-2.21) | | 0.430 | 0 (0-64) (0.80) | | 0.61-1.52 | | 0 | 0.27 | 1.00 | | 0.27 | | 1.00 | | 0.40 | 1.00 | |
| Nutrition and lifestyle | Torloni MR 2009 | Low vs. Normal BMI (cohort) | | | 16 | | 75669/280734 | | 0.75 (0.69-0.83) | | 0.77 (0.71-0.82) | | 0.80 (0.69-0.92) | | 0.022 | 16 (0-54) (0.27) | | 0.63-0.90 | | 6 | 11.2 | 0.01 | | 11.5 | | NP | | 10.3 | 0.03 | |
| Nutrition and lifestyle | Torloni MR 2009 | Low vs. Normal BMI (case-control) | | | 3 | | 5957/11651 | | 0.65 (0.51-0.83) | | 0.65 (0.51-0.82) | | 0.61 (0.47-0.81) | | 0.572 | 0 (0-73) (0.83) | | 0.13-3.16 | | 1 | 1.78 | 0.57 | | 1.78 | | 0.57 | | 1.96 | 0.28 | |
| Nutrition and lifestyle | Torloni MR 2009 | Overweight vs. Normal BMI (cohort) | | | 17 | | 112880/282458 | | 1.97 (1.76-2.19) | | 2.05 (1.94-2.15) | | 2.29 (2.12-2.47) | | 0.521 | 56 (11-73) (0.003) | | 1.44-2.68 | | 11 | 16.2 | NP | | 16.0 | | NP | | 16.5 | NP | |
| Nutrition and lifestyle | Torloni MR 2009 | Overweight vs. Normal BMI (case-control) | | | 3 | | 287/501 | | 2.68 (1.78-4.04) | | 2.66 (1.95-3.63) | | 3.85 (2.30-6.47) | | 0.889 | 40 (0-82) (0.19) | | 0.05-138 | | 3 | 2.86 | 1.00 | | 2.86 | | 1.00 | | 2.99 | 1.00 | |
| Nutrition and lifestyle | Torloni MR 2009 | Obese (BMI >30) vs. normal weight | | | 31 | | 56333/308335 | | 3.76 (3.31-4.28) | | 3.94 (3.75-4.13) | | 4.80 (4.43-5.21) | | 0.661 | 73 (60-80) (<0.001) | | 2.23-6.34 | | 26 | 31.0 | NP | | 31.0 | | NP | | 31.0 | NP | |
| Nutrition and lifestyle | Torloni MR 2009 | Obese 1 (BMI ~30–35) vs. Normal weight | | | 6 | | 3087/20901 | | 3.01 (2.34-3.86) | | 3.06 (2.62-3.56) | | 3.21 (2.68-3.85) | | 0.612 | 27 (0-71) (0.23) | | 1.71-5.28 | | 5 | 5.79 | 0.19 | | 5.78 | | 0.20 | | 5.83 | 0.16 | |
| Nutrition and lifestyle | Torloni MR 2009 | Obese 2 (BMI >35) vs. Normal weight | | | 7 | | 1747/21001 | | 5.52 (4.28-7.11) | | 5.42 (4.36-6.73) | | 5.10 (3.18-8.19) | | 0.157 | 7 (0-61) (0.37) | | 3.62-8.42 | | 6 | 6.98 | 0.02 | | 6.98 | | 0.02 | | 6.96 | 0.03 | |
| Nutrition and lifestyle | Torloni MR 2009 | Overweight vs. Non-overweight (cohort) | | | 34 | | 174233/391991 | | 2.95 (2.68-3.24) | | 2.81 (2.71-2.91) | | 3.10 (2.91-3.31) | | 0.132 | 72 (60-80) <0.001) | | 1.97-4.41 | | 31 | 33.9 | NP | | 33.9 | | NP | | 34.0 | NP | |
| Nutrition and lifestyle | Torloni MR 2009 | Overweight vs. Non-overweight (case-control) | | | 10 | | 6214/19567 | | 3.78 (2.49-5.76) | | 3.03 (2.68-3.42) | | 3.06 (2.51-3.73) | | 0.248 | 90 (84-93) (<0.001) | | 0.83-17.2 | | 9 | 9.65 | 0.30 | | 9.87 | | 0.13 | | 9.67 | 0.29 | |
| Nutrition and lifestyle | Torloni MR 2009 | Obese vs. non-obese (cohort) | | | 40 | | 68013/520879 | | 3.36 (3.01-3.74) | | 3.27 (3.14-3.41) | | 3.44 (3.20-3.70) | | 0.724 | 77 (68-82) (<0.001) | | 1.97-5.72 | | 36 | 40.0 | NP | | 40.0 | | NP | | 40.0 | NP | |
| Nutrition and lifestyle | Torloni MR 2009 | Obese vs. non-obese (case-control) | | | 3 | | 238/922 | | 3.24 (1.28-8.19) | | 3.36 (2.48-4.55) | | 7.49 (4.58-12.27) | | 0.938 | 88 (52-94) (0.001) | | 0-285401 | | 2 | 2.92 | 0.08 | | 2.9 | | 0.10 | | 3.00 | NP | |
|  |  |  | | |  | |  | |  | |  | |  | |  |  | |  | |  |  |  | |  | |  | |  |  | |
| Diseases/disorders | Pérez-López FR 2018 | Endometriosis | | | 12 | | 1973/46789 | | 1.14 (0.86-1.51) | | 1.14 (0.97-1.33) | | 0.81 (0.53-1.25) | | 0.642 | 56 (0-75) (0.009) | | 0.51-2.54 | | 1 | 1.69 | 1.00 | | 1.74 | | 1.00 | | 2.87 | 0.32 | |
| Diseases/disorders | Li L 2018 | Obstructive sleep apnea | | | 8 | | 18129/56707166 | | 1.71 (1.23-2.38) | | 1.74 (1.58-1.92) | | 1.89 (1.67-2.14) | | 0.961 | 83 (64-89) (<0.001) | | 0.64-4.62 | | 3 | 5.42 | 0.12 | | 5.35 | | 0.13 | | 5.75 | 0.04 | |
| Diseases/disorders | Li L 2018 | Snoring | | | 18 | | 2301/14216 | | 2.14 (1.63-2.81) | | 1.79 (1.56-2.06) | | 6.3 (3.77-10.53) | | 0.015 | 65 (36-78) (<0.001) | | 0.83-5.52 | | 8 | 11.6 | 0.09 | | 13.9 | | NP | | 17.9 | NP | |
| Diseases/disorders | Li L 2018 | Sleep-disordered breathing | | | 26 | | 20430/56721382 | | 1.95 (1.60-2.37) | | 1.76 (1.62-1.90) | | 1.89 (1.67-2.14) | | 0.180 | 72 (56-80) (<0.001) | | 0.90-4.22 | | 11 | 16.1 | 0.05 | | 18.0 | | 0.00 | | 17.5 | 0.01 | |
| Diseases/disorders | Gong LL 2016 | Overt hypothyroidism | | | 3 | | 3444/222161 | | 2.44 (1.08-5.52) | | 1.90 (1.69-2.14) | | 1.88 (1.67-2.12) | | 0.688 | 57 (0-86) (0.10) | | 0-15039 | | 2 | 2.20 | 1.00 | | 2.68 | | 0.29 | | 2.17 | 1.00 | |
| Diseases/disorders | Gong LL 2016 | Subclinical hypothyroidism | | | 6 | | 1859/61708 | | 1.59 (1.32-1.92) | | 1.59 (1.32-1.92) | | 1.49 (1.04-2.13) | | 0.208 | 0 (0-61) (0.50) | | 1.22-2.07 | | 3 | 5.26 | 0.03 | | 5.26 | | 0.03 | | 5.00 | 0.06 | |
| Diseases/disorders | Gong LL 2016 | Hypothyroidism (all) | | | 7 | | 5770/278609 | | 1.72 (1.51-1.95) | | 1.76 (1.60-1.94) | | 1.88 (1.67-2.12) | | 0.137 | 14 (0-64) (0.32) | | 1.35-2.18 | | 5 | 6.69 | 0.04 | | 6.64 | | 0.05 | | 6.78 | 0.02 | |
| Diseases/disorders | Kjerulff LE 2011 | Polycystic ovary syndrome | | | 18 | | 2385/89669 | | 2.83 (1.95-4.10) | | 2.68 (2.36-3.05) | | 2.69 (2.33-3.11) | | 0.653 | 52 (5-71) (0.005) | | 0.94-8.46 | | 8 | 14.9 | NP | | 15.4 | | NP | | 14.9 | NP | |
|  |  |  | | |  | |  | |  | |  | |  | |  |  | |  | |  |  |  | |  | |  | |  |  | |
| Infections | Abariga SA 2016 | Periodontitis | | | 10 | | 624/5100 | | 1.66 (1.16-2.36) | | 1.48 (1.17-1.87) | | 1.73 (0.91-3.30) | | 0.008 | 51 (0-74) (0.03) | | 0.61-4.49 | | 3 | 2.72 | 0.74 | | 4.03 | | 0.75 | | 4.54 | 0.33 | |
| Infections | Soepnel LM 2016 | HIV infection | | | 4 | | 593/1070 | | 0.83 (0.48-1.42) | | 0.83 (0.48-1.42) | | 1.00 (0.37-2.71) | | 0.472 | 0 (0-68) (0.61) | | 0.25-2.71 | | 0 | 0.67 | 1.00 | | 0.67 | | 1.00 | | 0.20 | 1.00 | |
|  |  |  | | |  | |  | |  | |  | |  | |  |  | |  | |  |  |  | |  | |  | |  |  | |
| Other | Xu Y-h 2018 | Extreme sleep duration | | | 12 | | 2602/37140 | | 1.43 (1.16-1.75) | | 1.34 (1.18-1.52) | | 1.29 (1.09-1.52) | | 0.039 | 33 (0-65) (0.12) | | 0.88-2.32 | | 5 | 4.75 | 1.00 | | 5.71 | | 0.78 | | 4.16 | 0.76 | |
| Other | Wang JW 2018 | Smoking vs non-smoking | | | 13 | | 22811/1341657 | | 0.98 (0.88-1.10) | | 0.96 (0.91-1.02) | | 0.90 (0.81-1.00) | | 0.634 | 49 (0-72) (0.02) | | 0.73-1.32 | | 3 | 1.70 | 0.24 | | 0.88 | | 0.05 | | 4.39 | 0.56 | |
| Other | Wang JW 2018 | Light smoking vs non-smoking | | | 5 | | 31257/1482334 | | 1.10 (0.97-1.24) | | 1.13 (1.06-1.20) | | 1.11 (1.02-1.21) | | 0.478 | 57 (0-82) (0.05) | | 0.77-1.58 | | 3 | 2.88 | 1.00 | | 2.63 | | 1.00 | | 2.71 | 1.00 | |
| Other | Wang JW 2018 | Heavy smoking vs non-smoking | | | 5 | | 17701/1064297 | | 1.02 (0.68-1.54) | | 0.90 (0.81-0.99) | | 0.90 (0.81-1.00) | | 0.534 | 60 (48-91) (0.04) | | 0.31-3.38 | | 2 | 1.81 | 1.00 | | 0.44 | | 0.06 | | 1.80 | 1.00 | |
| Other | Xiao Y 2018 | Age at menarche | | | 5 | | 2783/45752 | | 1.36 (1.15-1.60) | | 1.36 (1.20-1.53) | | 1.34 (1.14-1.58) | | 0.860 | 33 (0-75) (0.20) | | 0.88-2.10 | | 4 | 4.05 | 1.00 | | 4.04 | | 1.00 | | 3.95 | 1.00 | |
| Other | Moosazadeh M 2016 | Family history of diabetes | | | 33 | | 2697/29134 | | 3.46 (2.80-4.27) | | 3.50 (3.17-3.86) | | 4.36 (2.89-6.58) | | 0.861 | 76 (66-82) (<0.001) | | 1.17-10.2 | | 25 | 32.2 | NP | | 32.2 | | NP | | 16.1 | 0.00 | |
| Other | Xu Y 2016 | Isolated Single Umbilical Artery | | | 7 | | 1880/490712 | | 1.38 (1.06-1.80) | | 1.43 (1.17-1.75) | | 2.08 (1.47-2.96) | | 0.569 | 35 (0-72) (0.16) | | 0.73-2.61 | | 1 | 4.96 | NP | | 4.54 | | 0.01 | | 6.86 | NP | |
| Other | Pandey S 2012 | IVF/ICSI versus spontaneous conception | | | 6 | | 13399/574391 | | 1.31 (0.98-1.75) | | 1.50 (1.34-1.68) | | 1.55 (1.37-1.75) | | 0.169 | 42 (0-76) (0.13) | | 0.63-2.72 | | 1 | 4.26 | 0.01 | | 3.22 | | 0.10 | | 4.48 | NP | |
|  |  | |  |  | |  | |  | |  | |  | |  | | |  |  |  | |  | |  | |  | |  | | |  |

Abbreviations: Random effects, summary odds ratio (95% CI) using random effects model; Largest effect, odds ratio (95% CI) of the largest study in the meta-analysis; Egger, p-value from Egger's regression asymmetry test for evaluation of publication bias; P, p-value; NP, not pertinent, because the estimated is larger than the observed, and there is no evidence of excess of statistical significance based on the assumption made for the plausible effect size; BMI, Body Mass Index; GDM, gestational diabetes mellitus; PA, physical activity.

* Summary random effects odds ratio (95% CI) of each meta-analysis, except for three meta-analyses (Fu S 2016, Aune D 2016, Pandey S 2012 and Xiao Y 2018) where the RR was used.

† Summary fixed effects odds ratio (95% CI) of each meta-analysis, except for three meta-analyses (Fu S 2016, Aune D 2016, Pandey S 2012 and Xiao Y 2018) where the RR was used.

‡ Odds ratio (95% CI) of the largest study in each meta-analysis, except for three meta-analyses (Fu S 2016, Aune D 2016, Pandey S 2012 and Xiao Y 2018) where the RR was used.

§ P-value from the Egger regression asymmetry test for evaluation of publication bias

|| I^2^ metric of inconsistency (95% confidence intervals of I^2^) and P-value of the Cochran Q test for evaluation of heterogeneity

**≠** 95% Prediction Interval

¶ Observed number of statistically significant studies

# Expected number of statistically significant studies using the summary fixed effects estimate of each meta-analysis as the plausible effect size

** P-value of the excess statistical significance test

¥ Expected number of statistically significant studies using the summary random effects estimate of each meta-analysis as the plausible effect size

ȣ Expected number of statistically significant studies using the effect of the largest study of each meta-analysis as the plausible effect size
